# Investigations of Shape Deformation Behaviors of the Ferromagnetic Ni–Mn–Ga Alloy/Porous Silicone Rubber Composite towards Actuator Applications

**DOI:** 10.3390/mi14081604

**Published:** 2023-08-14

**Authors:** Wan-Ting Chiu, Yui Watanabe, Masaki Tahara, Tomonari Inamura, Hideki Hosoda

**Affiliations:** Institute of Innovative Research (IIR), Tokyo Institute of Technology, 4259 Nagatsuta-cho, Midori-ku, Yokohama 226-8503, Japan; yw.dandelion@gmail.com (Y.W.); tahara.m.aa@m.titech.ac.jp (M.T.); inamura.t.aa@m.titech.ac.jp (T.I.); hosoda.h.aa@m.titech.ac.jp (H.H.)

**Keywords:** actuators, composite material, ferromagnetic shape memory alloy, micromachines, martensite variant reorientation, Ni–Mn–Ga alloy, porous structure

## Abstract

Ferromagnetic shape memory alloys (FSMAs), which are potential candidates for future technologies (i.e., actuators in robots), have been paid much attention for their high work per volume and rapid response as external stimulation, such as a magnetic field, is imposed. Among all the FSMAs, the Ni–Mn–Ga-based alloys were considered promising materials due to their appropriate phase transformation temperatures and ferromagnetism. Nevertheless, their intrinsic embrittlement issue and sluggish twin motion due to the inhibition of grain boundaries restrict their practicability. This study took advantage of the single-crystal Ni–Mn–Ga cube/silicone rubber composite materials to solve the two aforementioned difficulties. The single-crystal Ni–Mn–Ga cube was prepared by using a high-temperature alloying procedure and a floating-zone (FZ) method, and the cubes were verified to be the near-{100}_p_ Ni–Mn–Ga alloy. Various room temperature (RT) curing silicone rubbers were utilized as matrix materials. Furthermore, polystyrene foam particles (PFP) were used to provide pores, allowing a porous silicone rubber matrix. It was found that the elastic modulus of the silicone rubber was successfully reduced by introducing the PFP. Additionally, the magnetic field-induced martensite variant reorientation (MVR) was greatly enhanced by introducing a porous structure into the silicone rubber. The single-crystal Ni–Mn–Ga cube/porous silicone rubber composite materials are considered to be promising materials for applications in actuators.

## 1. Introduction

Actuators with a rapid response and high work per volume are important components in applications of robots, which are considered critical devices for the development of future technologies [1,2,3,4]. Among all the candidates that are suitable materials for serving as actuators, the Ni–Mn–Ga-based alloys and their related alloys, whose shape change could be driven by a magnetic field, stress, and thermal factors, are considered promising materials for practicing robot applications [5,6,7]. It has been reported that the Ni–Mn–Ga alloys and their related alloys achieved high work per volume and fast responses to external stimulation, such as a magnetic field [8,9,10]. Therefore, the Ni–Mn–Ga alloy was chosen as the target material in this study.

To obtain the shape deformation, two different mechanisms could be practiced. The first one is shape deformation strain from the phase transformation between the austenite phase (L2_1_) and the martensite phase [11,12,13], while the second one is shape deformation strain from the martensite variant reorientation (MVR) [14,15,16]. It is known that more effort should be focused on obtaining shape deformation in the materials using the former mechanism, while shape deformation originating from the later mechanism could be achieved relatively easily [11,12,13,14,15,16,17,18]. Under either mechanism, the shape deformation changes induced by applying a magnetic field is known as a “magnetic field-induced strain” (MFIS) [19,20]. In addition, it is necessary to impose ferromagnetism on the Ni–Mn–Ga alloys to allow magnetic field-induced shape deformation [21,22]. Therefore, the chemical composition of the Ni–Mn–Ga alloy was determined to be Ni_50_Mn_28_Ga_22_ (at.%) based on the literature, which shows the dependence of the electron-to-atom (*e*/*a*) ratio on the phase stability [23]. Hence, the ferromagnetic five-layer modulated (5M) martensite of the Ni_50_Mn_28_Ga_22_ alloy was utilized in this study.

As aforementioned, Ni–Mn–Ga-based alloys and their related alloys are promising materials for use in robot applications; however, the embrittlement issue severely limits their applications [24,25]. In addition, it has been reported that the polycrystalline Ni–Mn–Ga alloys barely perform shape deformation brought about by the twin motions due to the constraint of the grain boundaries [26,27,28]. To solve the aforementioned two fundamental difficulties, the integration of the single-crystal Ni–Mn–Ga alloys with the polymer as composite materials could be a promising strategy [29,30,31]. It has been reported that the shape deformation of the composite materials could be as high as 4%, while the embrittlement and grain boundary constraint problems could be solved [31]. A series of studies on the composite materials composed of the single-crystal Ni–Mn–Ga alloys and silicone rubber was thus carried out in this study.

Concerning the selection of the polymers, to obtain the overall shape deformation of the composite material, some prerequisites should be considered. First, it is necessary to consider the Hardness Shore A of the polymer; once the elastic constraint of the polymer is greater than that of the stress of the MVR of the single-crystal Ni–Mn–Ga, no shape deformation could be achieved. Reversely, if the polymer is too soft, the transmission of stress inside the polymer will be inefficient; as a result, the overall shape deformation of the composite materials cannot be obtained either. Second, it is necessary to consider the curing temperature of the polymer since the phase stability of the shape memory alloys is highly sensitive to temperature. In particular, the phase transformation temperatures of the selected Ni–Mn–Ga alloys in this study are close to room temperature (i.e., 296 ± 3 K) [29,30,31,32]. A polymer that can be cured at RT under ambient conditions is thus demanded. Therefore, RT-cured silicone rubbers with various Hardness Shore A values were selected in this study for conducting the integration of the single-crystal Ni–Mn–Ga alloys.

To date, various composite materials of single-crystal Ni–Mn–Ga alloys/polymers have been studied [29,30,31]; however, only the solid silicone matrix is studied. It is expected that with the introduction of a porous structure to the silicone rubber, the MVR of the single-crystal Ni–Mn–Ga alloy could be enhanced, since the constraint of the polymer on the MVR of the single-crystal Ni–Mn–Ga alloy at the interface could be relieved by the existence of pores (i.e., free surface for the twin motions). It has been reported that surface conditions could also have a great influence on the MVR of the Ni–Mn–Ga alloys, since the pinning of the twin motion occurs at the surface or interface [33,34]. Hence, single-crystal Ni–Mn–Ga alloy/porous silicone rubber composite materials with various designs were examined in this work.

Polystyrene foam particles (PFP) were introduced into the silicone rubber to serve as pores in the silicone rubber matrix. It was found that the elastic modulus, which is a critical factor to the MVR of the single-crystal Ni–Mn–Ga alloy, was reduced with the increase in the pore volume percentage, showing a good trend. Additionally, more alloy surfaces could be freed with a higher pore volume percentage. Hence, the commencement and proceeding of the MVR could be promoted by increasing the volume percentage of the pores. For the purpose of comparison, solid silicone rubbers with various Hardness Shore A values were also examined. Some quantitative analyses of the MVR of single-crystal Ni–Mn–Ga alloys were also carried out. It was found that the porous silicone rubber could be a promising material for further study in this research series.

## 2. Materials and Methods

### 2.1. Alloy Preparations

As mentioned in the introduction section, for obtaining magnetic field-induced martensite variant reorientation (MVR), a composition of Ni_50_Mn_28_Ga_22_ (at.%) that was confirmed to be a single five-layer modulated (5M) martensite phase with ferromagnetism was selected [29,35]. The Ni_50_Mn_28_Ga_22_ (at.%) alloy is abbreviated as “Ni–Mn–Ga alloy” in the entire article unless otherwise mentioned.

High-purity nickel spheres (Ni; 99.99%, Kojundo Chemical Lab. Co., Ltd., Saitama, Japan), manganese flakes (Mn; 99.9%, Kojundo Chemical Lab. Co., Ltd., Saitama, Japan), and gallium spheres (Ga; 99.9999%, HIRANOSEIZAEMONSYOUTEN Co., Ltd., Nara, Japan) were used for the fabrication of the ingots. The Ni spheres and Mn flakes were subjected to solution cleansing using solutions of the HNO_3_:pure water = 1:1 (vol.%) and HNO_3_:pure water = 1:9 (vol.%), respectively. The Ga spheres were used as-received. The aforementioned raw materials were used as the starting materials for the high-temperature alloying processes by using the arc-melting system. The arc-melting system is equipped with a non-consumable tungsten electrode, and the chamber was filled with high-purity Ar + 1 vol.% H_2_ atmosphere (Kayama Oxygen Co., Ltd., Tokyo, Japan) during the entire high-temperature alloying process. The ingots were re-melted five times and they were flipped upside-down before each re-melting to achieve high homogeneity of the chemical composition. The ingots that were subjected to high-temperature alloying were then mechanically cleaned followed by solution cleansing. The ingots are denoted as “as-casted alloys”. To obtain the further homogenization of the chemical composition, the as-casted alloys were thereafter subjected to a homogenization process at 1273 K for 1 h followed by ice-water quenching. The homogenized alloys are denoted as “HT alloys” in the following sections.

### 2.2. Fabrication of Single-Crystal Ni–Mn–Ga Alloys

The HT alloys were mechanically polished and then cleaned using a solution. These alloys were utilized as starting materials for the fabrications of the single-crystal Ni–Mn–Ga alloys by using the floating-zone (FZ) method. For the details of the FZ method, please refer to our previous articles [36,37]. In brief, the feed ingot and seed ingot approached each other in a melting state and were combined into one. The upper and bottom shafts used for grabbing the feed and seed ingots were rotated at a speed of 30 rounds min^−1^ and were moved at a speed of around 5 mm h^−1^. During the entire FZ process, the FZ chamber was filled with a high-purity Ar gas at a pressure of around 0.4 MPa, while the flow rate of the Ar inlet was 0.2 L min^−1^. The material obtained via the FZ method is denoted as a “single-crystal Ni–Mn–Ga stick”.

### 2.3. Fabrication of Single-Crystal Ni–Mn–Ga Alloys/Polymer Composite Materials

For the fabrication of the composite materials, single-crystal Ni–Mn–Ga cubes with dimensions of 1 mm × 1 mm × 1 mm and 2 mm × 2 mm × 2 mm, respectively, were used; alloys with a cube structure were sliced off of the FZ method-treated single-crystal Ni–Mn–Ga stick by an electrical discharge machine (EDM). Prior to the slicing of the single-crystal Ni–Mn–Ga cubes, the crystallographic direction was firstly verified. The surfaces of the single-crystal Ni–Mn–Ga cube specimens were designated to be in the near-{100}_p_ plane. The subscript “p” indicates the parent phase of the austenite phase. For details of the confirmation of crystallographic direction, please refer to our previous publications [36,37,38,39]. The single-crystal Ni–Mn–Ga cube specimens were thereafter mechanically ground and integrated with the polymers.

For the fabrication of composite materials, different types of polymers have been used. Silicone rubbers with Hardness Shore A values of 23 (ELASTOSILM M4400, Wacker Chemie AG, Munich, Germany), 27 (ELASTOSILM M8520, Wacker Chemie AG), 35 (ELASTOSILM SLJ3220, Wacker Chemie AG), 45 (ELASTOSILM M8012, Wacker Chemie AG), 50 (ELASTOSILM M8017, Wacker Chemie AG), and 60 (ELASTOSILM M4470, Wacker Chemie AG) were used in this study, respectively. The aforementioned silicone rubbers are summarized in Table 1(a). The corresponding curing agent (Catalyst T40, Wacker Chemie AG) was used for all the silicone monomers except for the silicone rubber with the Hardness Shore A of 35, while a corresponding curing agent (Catalyst T47, Wacker Chemie AG) was utilized for the silicone monomer with the Hardness Shore A of 35. In the case of the M4400 and M4470 silicone rubbers, a ratio of silicone monomer:curing agent = 100:3 (wt.%) was used. On the other hand, in the case of the other silicone rubbers, a ratio of silicone monomer:curing agent = 100:4 (wt.%) was used. The mixture of the silicone monomer and its corresponding curing agent was then well-mixed at room temperature (RT; i.e., 296 K ± 3K) using a hybrid mixer (HM-500, KEYENCE, Osaka, Japan). The mixture was subjected to the mixing program of mixing for 1 min followed by degassing for 1 min, and the resulting well-mixed semi-liquid polymer is denoted as “slurry”.

The slurry was then integrated with the single-crystal Ni–Mn–Ga cube, which was finely mechanically polished in advance. Concerning the fabrication processes of the various composite materials, please refer to other articles [36,37,38,39]. After the integration of the single-crystal Ni–Mn–Ga cube and the polymer, the polymer matrix was trimmed to obtain certain volume percentages of the single-crystal Ni–Mn–Ga cube in the silicone rubber matrix.

It has been reported that a disappearing or deteriorated twinning deformation occurs when the stiffness of the surrounding silicone rubber matrix is higher than a specific critical point. In other words, the stress that is required for the twin motion of the single-crystal Ni–Mn–Ga cube is smaller than the elastic constraint generated by the silicone rubber matrix [36,37,38,39,40]. In addition, again, the surface condition also has an influence on the MVR. Therefore, besides the aforementioned various elastic modulus values of the silicone rubber, in this study, a silicone rubber matrix with a porous structure was considered and was carried out. Polystyrene foam particles (PFP; HKB-P, SEKIZUKA Co., Ltd., Sukagawa, Japan) were used to provide pores in the silicone rubber. The silicone rubber with a Hardness Shore A of 50 (ELASTOSILM M8017, Wacker Chemie AG) was used for the examination of the effects of pores on the silicone matrix. Various volume percentages of PFP in the silicone rubber were designed and are shown in Table 1(b).

### 2.4. Measurements

In this measurement section, all specimens have been examined three times to confirm their reproducibility. One result has been chosen as a representative.

#### 2.4.1. Identification of Phase Constituent

An X-ray diffractometer (XRD; PANalytical X’Pert PRO MPD) in the arrangement of *θ–*2*θ* was used for phase identification at RT under ambient conditions. The HT alloys were mechanically crushed into particles followed by a heat-treatment of 1073 K for 2 h, and the heat-treated particles were then used as the specimen for phase identification. CuK_α_ radiation was used as the X-ray incident beam, while the tube voltage and the tube current were set at 45 kV and 40 mA, respectively. The *θ*–2*θ* scan was in the range of 2*θ* = 20–120° at the scan rate of 2.2° min^−1^.

#### 2.4.2. Analysis of Thermal Behaviors

For determining the phase transformation temperatures and the Curie temperature (*T*_c_) during both heating and cooling, differential scanning calorimetry (DSC; DSC-60 Plus, Shimadzu Corporation, Kyoto, Japan) was utilized. The HT alloys were utilized as testing specimens while the Al_2_O_3_ powders with a certain weight were used as reference materials. A high-purity Ar atmosphere was used during the thermal analysis for preventing the oxidation reaction of the specimens at an elevated temperature. The temperature scan was from 273 K to 383 K at a scan rate of 10 K min^−1^.

#### 2.4.3. Examinations of Elastic Modulus of Various Silicone Rubbers

Compression tests were used for the examination of the elastic modulus of various pure silicone rubbers (with or without pore structure) at RT under ambient conditions. For the compression tests, a universal testing machine (AUTOGRAPH AG-X plus, SHIMAZU, Kyoto, Japan) was used. The details of the settings and operations of the compression examinations can be found elsewhere [36,37,38,39]. The silicone rubbers, which were utilized in this work, are listed in Table 1(a). As mentioned previously, a porous structure was introduced into the silicone rubber by inserting the PFP at specific volume percentages (vol.%). Again, the vol.% of the silicone rubber to the PFP is also listed in Table 1(b), showing a total volume percentage of 100%.

The pure silicone rubbers (silicone rubbers with the Hardness Shore A from 23 to 60) were fabricated in the shape of a cylinder possessing a diameter of 11 mm (*x*-axis and *y*-axis) and a height of 17 mm (*z*-axis), respectively. The porous silicone rubbers (silicone rubber with a Hardness Shore A of 50) were fabricated in the shape of a square plate possessing dimensions of 10 mm (width) × 10 mm (length) × 2 mm (height).

In the compression examinations, the cylinder-structured solid silicone rubber specimens were compressed along their *z*-axis, which is also the height dimension, with a height of 17 mm. The compression examinations were conducted at a constant compression rate of 1 × 10^−3^ s^−1^. On the other hand, the plate-structured porous silicone rubber specimens were compressed in their height dimension (i.e., along the short side of the 2 mm side). Illustrations of these two compression examinations are shown in the stress–strain (*S*-*S*) curves in the results and discussion section. Prior to the compression examinations, a lubricant was applied to the specimen stage for reducing the friction between the specimen and the specimen stage. 

#### 2.4.4. Evaluations of Magnetic Properties

To evaluate the magnetic properties of the single-crystal Ni–Mn–Ga cube/silicone rubber composite materials, a vibrating sample magnetometer (VSM; TM-VSM1530-HGC-D, Tamakawa Co., Sendai, Japan) was used at RT under ambient conditions. A standard Ni cube was utilized as a reference material for calibration. The scan range and the scan rate were ± 10 kOe and 0.2 kOe s^−1^, respectively. Concerning the evaluations of magnetic properties, it is necessary to mention that it has been reported that the embedded single-crystal Ni–Mn–Ga cube showed limited dependence on small variations in the angle (within 10° difference) [38,39]. Therefore, deviations of the near-{100}_p_ single-crystal Ni–Mn–Ga cube used could almost be ignored.

#### 2.4.5. Observations of Microstructure

To verify the interface between the single-crystal Ni–Mn–Ga cube and the silicone rubber, optical microscopy (OM; VHX-100F, KEYENCE, Osaka, Japan) was utilized for the observation of the microstructure of the composite materials at RT under ambient conditions.

#### 2.4.6. Analysis of Deformation

To analyze the deformation of the composite materials under a certain magnetic field, a laser sensor (LS-5000, KEYENCE, Osaka, Japan) was utilized at RT under ambient pressure. (1) The single-crystal Ni–Mn–Ga cubes, (2) the single-crystal Ni–Mn–Ga cube/silicone rubber composites, and (3) the polycrystal Ni–Mn–Ga cubes/silicone rubber composites served as the testing specimens, respectively. The specimen (3) of the polycrystalline Ni–Mn–Ga cubes/silicone rubber composite materials were utilized for the calibration of the shift of the composite materials when a magnet was applied. Therefore, the compensated deformation could be attributed to merely the MVR of the single-crystal Ni–Mn–Ga cube.

The setting of the measurements is shown in Figure S1 in the Supplementary Materials. As shown in Figure S1, the specimen was firstly placed on the top of the stage without the introduction of a magnet (*H*-field = 0 kOe). Secondly, a magnet was then attached to the ceiling of the stage (*H*-field = 4.3 kOe). Lastly, the magnet was removed from the ceiling of the stage (*H*-field = 0 kOe). The deformations in the above-mentioned three different states of the specimens were measured by applying a laser, as shown in the illustration (Figure S1c). The top of the specimen was used as the starting point for the deformation analysis.

## 3. Results and Discussion

### 3.1. Identification of Phase Constituent

**Figure 1:**
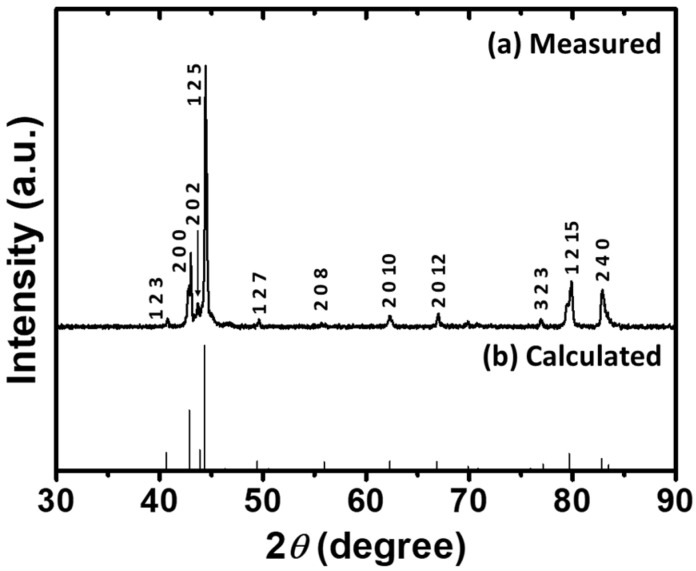
The (**a**) measured and (**b**) calculated X-ray diffraction patterns of the HT alloy of the Ni_50_Mn_28_Ga_22_ specimen.

The polycrystalline particles, which were derived from mechanically crushing the HT alloys, served as the testing specimens for phase identification. In Figure 1, both the (a) measured and (b) calculated X-ray diffraction patterns are shown, respectively. Please note that the (b) calculated pattern was based on the software of CaRIne crystallography version 3.1. It was thus confirmed that the designated Ni_50_Mn_28_Ga_22_ (at.%) was composed of the single 5M-martensite phase at RT under ambient conditions. This phase identification is also consistent with that shown in the literature [41,42].

### 3.2. Analysis of Thermal Behaviors

**Figure 2:**
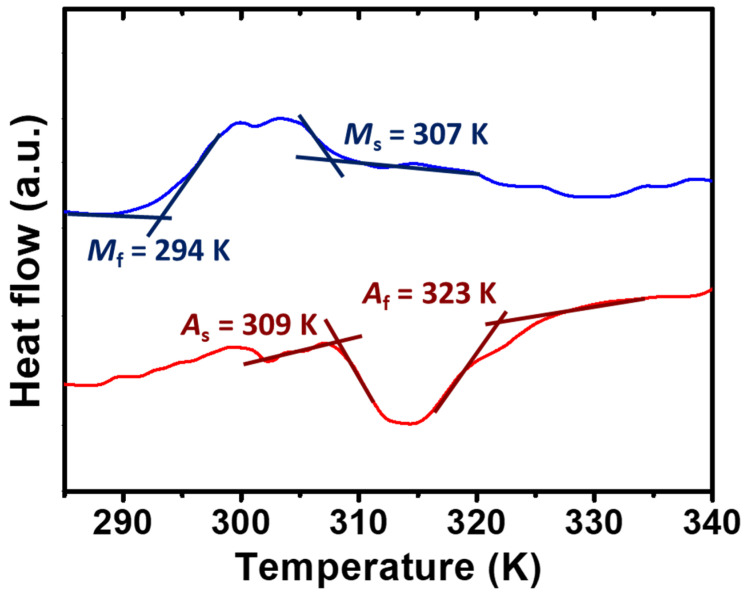
The heating and cooling curves of the HT alloys. (*A*_s_: reverse martensitic transformation start temperature; *A*_f_: reverse martensitic transformation finish temperature; *M*_s_: forward martensitic transformation start temperature; *M*_f_: forward martensitic transformation finish temperature).

To determine the phase transformation temperatures and the phase constituent at RT, thermal analysis was conducted on the HT alloys. The heating and cooling curves are shown in Figure 2. The abbreviations in Figure 2 are as follows: *A*_s_—reverse martensitic transformation start temperature; *A*_f_—reverse martensitic transformation finish temperature; *M*_s_—forward martensitic transformation start temperature; *M*_f_—forward martensitic transformation finish temperature. The phase transformation temperatures were determined using the tangent method. The *A*_s_, *A*_f_, *M*_s_, and *M*_f_ were determined to be 309, 323, 307, and 294 K, respectively. Judging from the aforementioned phase transformation temperatures, it was concluded that the single-crystal Ni–Mn–Ga cubes, which were used as filler for the composite materials, were composed of the single-phase of the ferromagnetic 5M-martensite phase at RT under ambient pressure.

### 3.3. Evaluations of Elastic Modulus

Two different structures of the silicone rubbers were tested; the first type of specimen is a single solid silicone rubber without the introduction of PFP (i.e., non-porous structure), while the second type of specimen is a silicone rubber with the introduction of PFP, making it a porous structure. The elastic modulus values of the aforementioned two general types of specimens were tested by conducting compression examinations at RT under ambient conditions. Again, please note that the solid silicone rubber was in the shape of a cylinder, while the porous silicone rubber was in the shape of a plate, as mentioned in the experimental section previously.

**Figure 3:**
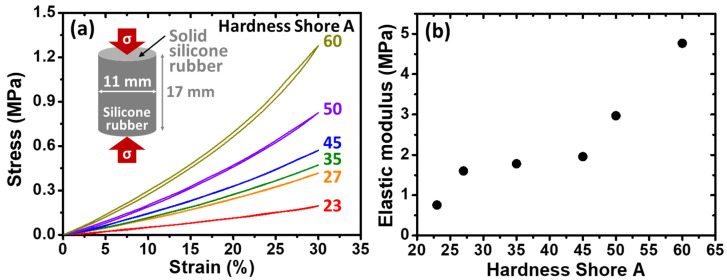
(**a**) The stress–strain (*S*-*S*) curves of the pure solid silicone rubbers with various Hardness Shore A values as listed in Table 1 (numbers next to the curves indicate the Hardness Shore A). An illustration of the cylinder-shaped testing specimen with a diameter of 11 mm and height of 17 mm, respectively, is inserted into (**a**). The red arrows with a symbol of *σ* suggest the compression direction. (**b**) The estimated elastic modulus of each solid silicone rubber from (**a**) as a function of Hardness Shore A (error bars are within the symbols).

The stress–strain (*S*-*S*) curves of the single solid silicone rubbers (i.e., without the introduction of the PFP) with different Hardness Shore A values are shown in Figure 3a, while the estimated elastic modulus values of each silicone rubber as a function of Hardness Shore A are shown in Figure 3b. An illustration of the testing specimen of pure solid silicone rubber is inserted into Figure 3a, showing a cylinder shape with a diameter of 11 mm (*x*-axis and *y*-axis) and height of 17 mm (*z*-axis), respectively. Please note that the elastic modulus was estimated based on the slope at an overall strain of around 20% due to the stability issue of the silicone rubber. It is clear that, as expected, the higher the Hardness Shore A, the larger the elastic modulus. In addition, the hysteresis of the pure solid silicone rubber increased with the Hardness Shore A.

**Figure 4:**
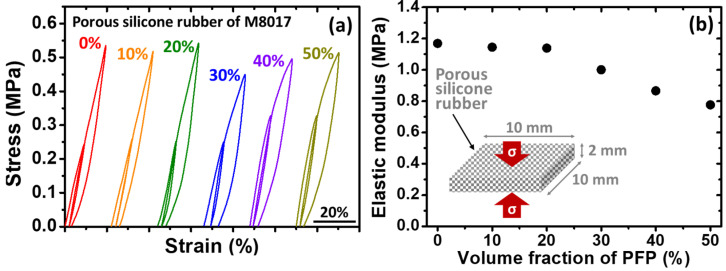
(**a**) The stress–strain (S-S) curves of the porous silicone rubbers (Hardness Shore A = 50; M8017 silicone rubber) with various pore volume percentages (vol.%) as listed in Table 1 (numbers next to the curves indicate the vol.% of pores). (**b**) The estimated elastic modulus of each porous silicone rubber from (**a**) as a function of pore vol.%. An illustration of the porous silicone rubber used as the testing specimen with a dimension of 10 mm × 10 mm × 2 mm is inserted into (**b**). The red arrows with a symbol of *σ* suggest the compression direction (error bars are shown within the symbols).

The *S*-*S* curves of the porous silicone rubber (with a Hardness Shore A of 50 (M8017 silicone rubber)) with various volume fractions of pores are shown in Figure 4a. The numbers adjacent to the curves indicate the volume fraction of the pore (i.e., the volume fraction of the PFP introduced). The elastic modulus of the porous silicone rubber specimens was read and estimated from the curves, and is plotted in Figure 4b. An illustration of the testing specimen is inserted into Figure 4b. To stabilize the porous silicone rubber, a pre-strain was introduced as the first cycle, and the second cycle was conducted followed by the first compression cycle. In Figure 4b, as expected, it is obvious that the elastic modulus of the porous silicone rubber decreased with the increase in the pore volume percentage. This indicates a softening of the specimens when the porous structure was introduced.

### 3.4. Observation of Martensitic Variant Reorientation (MVR)

#### 3.4.1. MVR Behaviors of the Single-Crystal Ni–Mn–Ga Cubes

**Figure 5:**
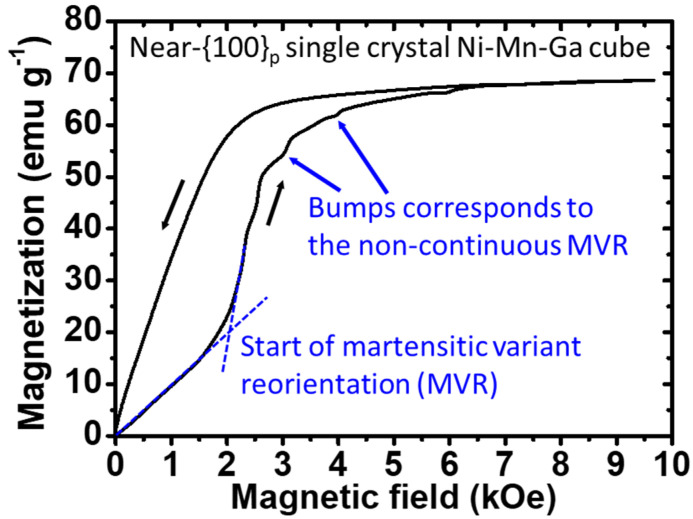
The *M*-*H* curve of the near-{100}_p_ single-crystal Ni–Mn–Ga cube at RT under ambient conditions. The crossing of the two dashed tangent lines indicates the commencement of the martensitic variant reorientation (MVR). Black arrows adjacent to the curves suggest the scanning direction, while blue arrows indicate the non-continuous MVR (i.e., a sudden jump in the magnetization).

For the observations of the martensitic variant reorientation (MVR), which could be triggered by the introduction of an external magnetic field, the single-crystal Ni–Mn–Ga cube was subjected to the scanning of a magnetic field from 0 to 10 kOe (Figure 5). A sudden jump in magnetization or an obvious change in the slope in the *M*-*H* curve after a certain critical magnetic field is reached has been reported. This indicates the start of the magnetic field-induced MRV and also its progress [36,37,38,39]. In other words, a sudden increase in the magnetization or a change in the slope of the curve could be observed when the start of the MVR takes place. Eventually, a saturation of the magnetization could be found when the MVR approached its end.

Therefore, based on the aforementioned things, first, the dashed tangent lines, which imply a sudden change in the slope (or the sudden change in the magnetization), are displayed in Figure 5, suggesting a commencement of MVR. Judging from Figure 5, it could be determined that the commencement of the magnetic field-induced MVR of the single-crystal Ni–Mn–Ga was around 2 kOe. This result is similar to others in the literature [35,36,37,38,39]. Second, followed by the jump in magnetization, it is observed that, in the *M*-*H* curve, before reaching saturation, there are some non-continuous bumps, which suggests the reorientation of the remaining variants (i.e., progressing of the MVR) in the single-crystal Ni–Mn–Ga cube, triggered by the magnetic field applied. Last, when the MVR approaches an end (i.e., the finish of the MVR), there is a saturation of magnetization, as shown in Figure 5. Therefore, the net increase in the amount of the magnetization brought about by the overall MVR from the crossing of tangent lines, as illustrated in Figure 5, to the saturation of magnetization corresponds to the sum of all sudden jumps (see the crossing of tangent lines and bumps in the figure) in magnetization (i.e., *y*-axis).

#### 3.4.2. MVR Behaviors of the Single-Crystal Ni–Mn–Ga Cubes/Solid Silicone Rubber

Besides the aforementioned measurements of the single-crystal Ni–Mn–Ga cubes, the MVR behaviors of the composite materials of the single-crystal Ni–Mn–Ga cubes/silicone rubber were also examined. Please notice that, as mentioned previously, various elastic modulus values of the solid silicone rubbers have been used for the integration of the composite materials. Besides this, in this section, the porous structure was not introduced into the silicone rubber matrix. The volume percentage of the single-crystal Ni–Mn–Ga cubes of the composite materials was about 6.5%. The MVR behaviors of the composite materials were examined and are shown as follows.

**Figure 6:**
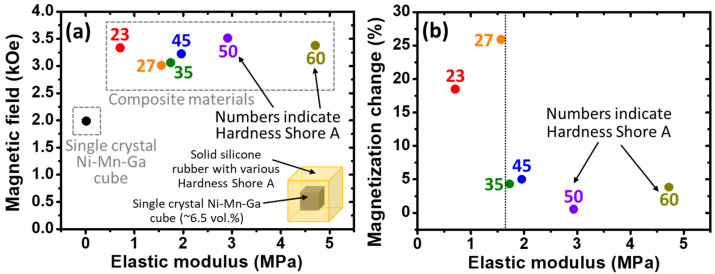
(**a**) The commencement of the MVR of the single-crystal Ni–Mn–Ga cube in the composite materials triggered by an externally applied magnetic field as a function of the elastic modulus of the various solid silicone rubber matrices. The examinations were conducted at RT under ambient conditions with a magnetic field scan range of 0 to 10 kOe. The dashed squares indicate the single-crystal and the composite materials, respectively, while the numbers adjacent to the dots suggest the Hardness Shore A of the various silicone rubber. An illustration is inserted to reveal the design of the composite materials. (**b**) The accumulated magnetization changes compared to the single-crystal Ni–Mn–Ga cube during the scan of the magnetic field at RT under ambient conditions as a function of the elastic modulus of the solid silicone rubber. The vertical dotted line indicates the critical elastic modulus of the solid silicone rubber. Error bars are within the symbols. The size of the Ni–Mn–Ga cube in the center of the composite was about 1 mm × 1 mm × 1 mm and the size of the silicone rubber was about 2.5 mm × 2.5 mm × 2.5 mm (i.e., ~6.5 vol.% of Ni–Mn–Ga cube).

Similar to the examination of the single-crystal Ni–Mn–Ga cube, the *M*-*H* curves of the composite materials were examined at RT under ambient pressure and the results of the elastic modulus are shown in Figure 6a. For each *M*-*H* curve measured, please refer to Figure S2 in the Supplementary Materials. The necessary magnetic field required for triggering the MVR of the single-crystal Ni–Mn–Ga in the composite materials is plotted as a function of the elastic modulus of the solid silicone rubber matrix. Please note that the necessary magnetic field for the MVR of the single-crystal Ni–Mn–Ga cube can also be read from Figure 5, and is plotted in Figure 6a (see the black dot at the elastic modulus = 0 MPa). In Figure 6a, the two dashed squares indicate the single-crystal Ni–Mn–Ga cube specimen and the composite materials, respectively, as denoted by the descriptions. The numbers adjacent to the dots suggest the Hardness Shore A value of each solid silicone rubber. An illustration of the composite material is also inserted into Figure 6a at the bottom-right corner to reveal the design of the composite materials.

It is obvious that with the integration of the solid silicone rubber as a matrix material, the necessary magnetic field increased from around 2 kOe to around 3.3 kOe (see the small dashed square and the large rectangle). The elevated magnetic field required is due to the constraint applied by the solid silicone rubber on the commencement of MVR of the single-crystal Ni–Mn–Ga cube. However, it seems that a variation in the elastic modulus did not affect the commencement of magnetic field-triggered MVR too much from around 0.6 to 5 MPa of the elastic modulus. That is, all the MVR values of the single-crystal Ni–Mn–Ga cubes integrated with different silicone rubbers remained around 3.3 kOe.

To further analyze the effects of the silicone rubber on the MVR behaviors, the accumulated magnetization change brought about by MVR during the magnetic field scan was estimated based on the *M*-*H* curves, and then was compared with the single-crystal Ni–Mn–Ga cube (Figure 6b). Here, please note that the term “accumulated magnetization change” refers only to the magnetization change brought about by the MVR (i.e., the sudden jump of the magnetization). Similar to Figure 6a, the numbers adjacent to the dots indicate the Hardness Shore A of each solid silicone rubber. It is necessary to mention that the accumulated magnetization changes are described in the unit of percentage attained when using the single-crystal cube as a reference material; that is, the single-crystal Ni–Mn–Ga cube is considered 100%. The equation could be written as:

(1) $$Magnetization\;change\left(\%\right)=\frac{\frac{Accmulated\;M\;changes\;of\;composite}{Saturation\;M\;of\;composite}}{\frac{Accmulated\;M\;changes\;of\;single\;crystal}{Saturation\;M\;of\;single\;crystal}}$$

where the symbol “*M*” indicates the magnetization. It is obvious that when the elastic modulus went beyond 1.6 MPa (vertical dotted line in Figure 6b), the accumulated magnetization change decreased greatly. On one hand, when the elastic modulus of the solid silicone rubber was lower than 1.6 MPa, the accumulated non-continuous increase in the magnetization brought about by the MVR was around 20–25%. On the other hand, when the elastic modulus of the solid silicone rubber was greater than 1.6 MPa, the accumulated non-continuous increase in the magnetization brought about by the MVR suddenly dropped to around 0–5%. Thus, it can be concluded that the critical elastic modulus of the solid silicone rubber required for the MVR of the single-crystal cubes is around 1.6 MPa.

According to Figure 6, it was found that the effect of the silicone rubber elastic modulus on the commencement of the MVR of the single-crystal Ni–Mn–Ga cube was slight (around 3.3 kOe for starting MVR); however, once the elastic modulus of the silicone rubber went beyond 1.6 MPa, the accumulated magnetization change of the single-crystal Ni–Mn–Ga cube brought about by the MVR decreased greatly. Therefore, it could be concluded that for the fabrication of composite materials, the elastic modulus of the silicone rubber should be designed to be less than 1.6 MPa in order to obtain the commencement of MVR and subsequent shape deformation.

#### 3.4.3. MVR Behaviors of the Single-Crystal Ni–Mn–Ga Cubes/Porous Silicone Rubber

**Figure 7:**
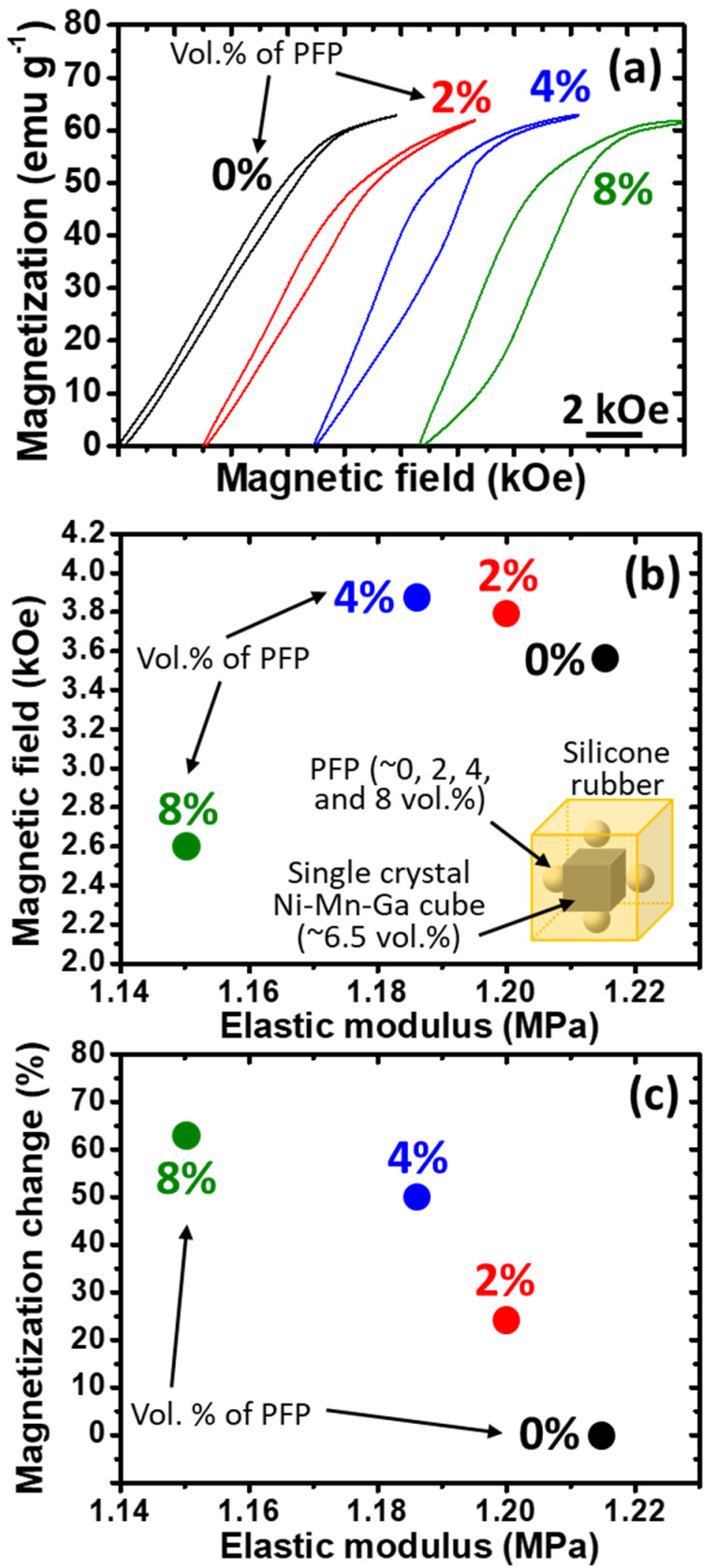
(**a**) The *M*-*H* curves of the composite materials of the single-crystal Ni–Mn–Ga cube/porous silicone rubber with the volume percentages of the PFP set at 0%, 2%, 4%, and 8%, respectively. The numbers adjacent to the dots indicate the vol.% of PFP. (**b**) The necessary magnetic field required for the commencement of MVR of the single-crystal Ni–Mn–Ga cube in the porous silicone rubber matrix with the PFP vol.% of 0%, 2%, 4%, and 8%, respectively, as a function of elastic modulus of the porous silicone rubber matrix. An illustration is inserted for revealing the design of the porous composite materials, while the numbers adjacent to the dots indicate the vol.% of PFP. (**c**) The accumulated magnetization changes in the single-crystal Ni–Mn–Ga cube in the porous silicone rubber matrix with the PFP vol.% of 0%, 2%, 4%, and 8%, respectively, as a function of elastic modulus of the porous silicone rubber matrix. Error bars are within the symbols. The size of the Ni–Mn–Ga cube in the center of the composite was about 1 mm × 1 mm × 1 mm and the size of the silicone rubber was about 2.5 mm × 2.5 mm × 2.5 mm (i.e., ~6.5 vol.% of Ni–Mn–Ga cube). The size of the PFP was about 600–700 μm.

Besides the aforementioned composite materials of the single-crystal Ni–Mn–Ga cubes/solid silicone rubber with various elastic modulus values, composite materials of the single-crystal Ni–Mn–Ga cubes/porous silicone rubber with various pore volume percentages were also investigated, and their results are shown in this section. Similar to Section 3.4.2, to reveal the magnetic field-induced MVR behaviors, the *M*-*H* measurements were carried out at RT under ambient conditions. The volume percentage of the single-crystal Ni–Mn–Ga cube, which was at approximately 6.5%, was identical to that in the previous section, while the volume percentages of the PFP were 0%, 2%, 4%, and 8%, respectively. The *M*-*H* curves are shown in Figure 7a, while the necessary magnetic field required for the commencement of MVR and the accumulated magnetization change of the single-crystal Ni–Mn–Ga cube can be read from Figure 7a and are plotted in Figure 7b,c, respectively. In all figures, the numbers adjacent to the curves and dots indicate the volume percentage of the introduced PFP, while an illustration of the porous composite material is inserted into Figure 7b.

From Figure 7a, it is obvious that the higher the volume percentage of the PFP, the lower the magnetic field required for the commencement of MVR of the single-crystal Ni–Mn–Ga cube. In addition, it is also found that the higher the PFP volume percentage, the larger the hysteresis. This could be attributed to the less back stress brought about by the silicone rubber in the high-PFP composite. For example, in the forward scan, MVR could take place under a relatively low magnetic field; however, in the reverse scan, a reduction in magnetization could not be provided by the surrounding silicone rubber due to the high percentage of PFP. Thus, a larger hysteresis was attained as the volume percentage of PFP increased. The necessary magnetic field required for the commencement of MVR of the single-crystal Ni–Mn–Ga cube can be read from Figure 7a, and is shown as a function of the elastic modulus of the porous silicone matrix (Figure 7b). An illustration is inserted in Figure 7b that reveals the design of the porous composite material. The elastic modulus values of the porous silicone rubbers shown in Figure 7 were estimated from the results shown in Figure 4. It is known that the greater the PFP volume percentage, the lower the elastic modulus (Figure 4). Hence, the MVR of the single-crystal Ni–Mn–Ga cube could be easily triggered by the applied magnetic field as the elastic modulus of the porous silicone rubber is reduced. The necessary magnetic field remained around 3.6 to 3.8 kOe, with a certain deviation when the volume percentage of the PFP ranged from 0% to 4%. On the other hand, the necessary magnetic field required for the commencement of the MVR of the single-crystal Ni–Mn–Ga cube was reduced to around 2.6 kOe when the volume percentage of the PFP was increased to 8%.

Accordingly, it is clear that with the introduction of the porous structure to the silicone rubber using the PFP, the commencement of MVR of the single-crystal Ni–Mn–Ga cube could be achieved relatively easily. Besides this, it seems that the critical volume percentage of the pore structure could be between 4% and 8%, while the elastic modulus was in the range of about 1.15 to 1.18 MPa. Based on the aforementioned observations, the enhanced MVR of the single-crystal Ni–Mn–Ga cube could be attributed to both the lower elastic modulus and the porous structure. Further discussions are shown in the following sections.

The accumulated magnetization changes of each specimen are also shown in Figure 7c as a function of the elastic modulus of the porous silicone matrix. As expected, the higher the vol.% of the PFP (or the lower the elastic modulus of the porous silicone rubber matrix), the more the accumulated magnetization changes. It was found that without the introduction of PFP, no accumulated magnetization changes could be observed; however, with the introduction of 8 vol.% PFP, the accumulated magnetization changes achieved were as high as 60% compared to the single-crystal Ni–Mn–Ga cube (used as the reference of 100% accumulated magnetization changes). It is clear that with the introduction of a porous structure to the silicone rubber, accumulated magnetization changes could be achieved relatively easily.

#### 3.4.4. Effect of PFP Configuration on the MVR of the Single-Crystal Ni–Mn–Ga Cube

**Figure 8:**
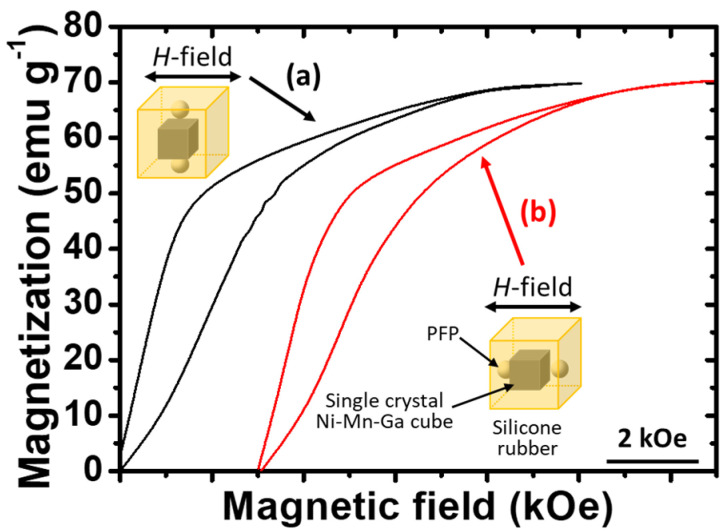
Effect of the configuration of PFP in the silicone rubber matrix on the MVR of the single-crystal Ni–Mn–Ga cube with the alignments of (**a**) PFP vertical to the *H*-field and (**b**) PFP parallel to the *H*-field. The corresponding *M*-*H* curves are indicated by arrows. The size of the Ni–Mn–Ga cube in the center of the composite was about 1 mm × 1 mm × 1 mm and the size of the silicone rubber was about 2.5 mm × 2.5 mm × 2.5 mm (i.e., ~6.5 vol.% of Ni–Mn–Ga cube). The size of the PFP was about 600–700 μm.

To reveal the effects of the configuration of the PFP in the porous silicone rubber matrix on the MVR of the single-crystal Ni–Mn–Ga cube, two different configurations were employed, and their configurations and *M*-*H* curves are shown in Figure 8. Figure 8a shows an illustration and the *M*-*H* curve derived when the PFP alignment is vertical to the applied *H*-field. On the other hand, Figure 8b shows the illustration and the *M*-*H* curve for the PFP alignment parallel to the applied *H*-field.

Judging from Figure 8a,b, it could be concluded that there is only a limited effect of the PFP configuration in the porous silicone rubber matrix on the MVR of the single-crystal Ni–Mn–Ga cube. According to the aforementioned results, it could be assumed that the influence of the volume fraction of the PFP in the silicone rubber is greater than that of the configuration of the PFP.

In brief, as the volume fraction of the PFP increased, the reduced elastic modulus and the pore structure (i.e., a freed interface between the PFP and the cube) of the porous silicone rubber could facilitate the MVR of the single-crystal Ni–Mn–Ga cube; on the other hand, the effect of the configuration of the introduced PFP on the MVR of the single-crystal Ni–Mn–Ga cube could almost be ignored.

### 3.5. Quantitively Analysis of MVR

**Figure 9:**
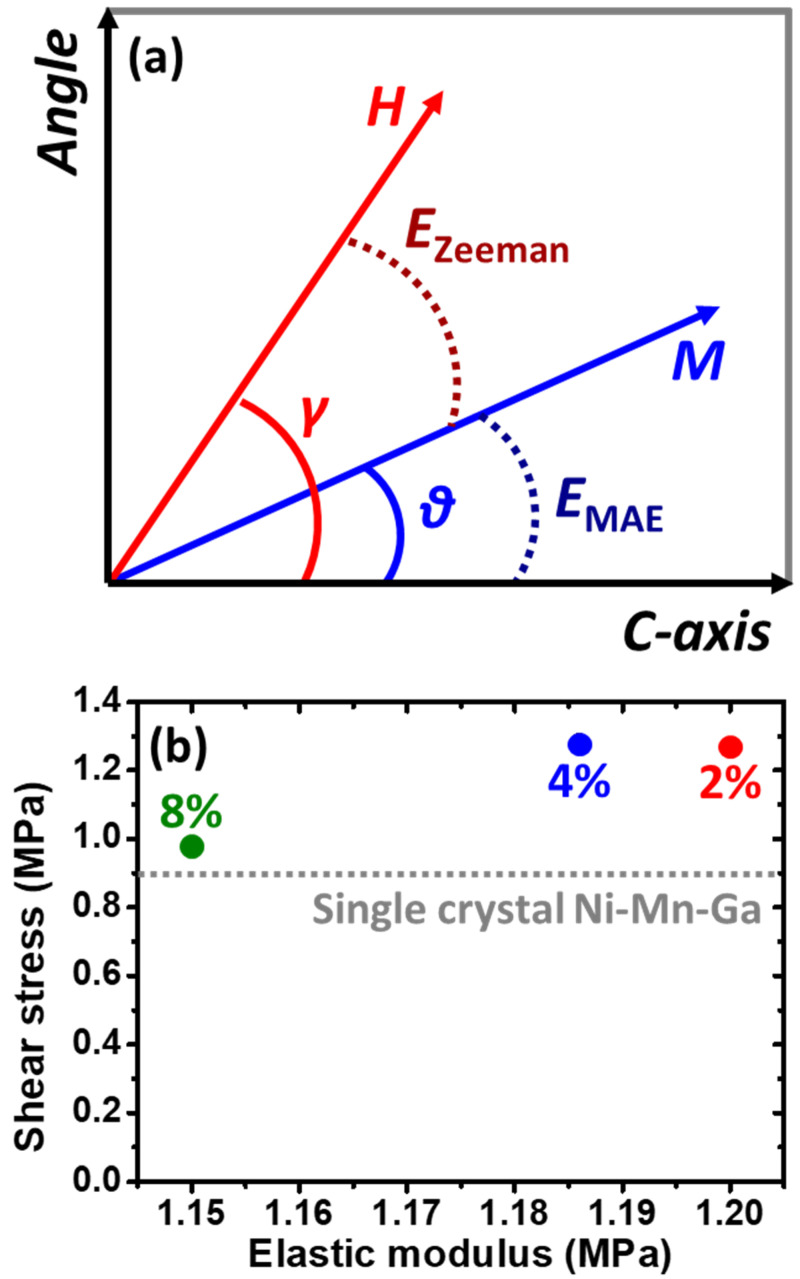
(**a**) The illustration reveals the relationships among the magnetic field direction applied, magnetization, and the easy axis of the *c*-axis of the Ni–Mn–Ga alloy (where, *M* = magnetization, *H* = magnetic field, *γ* = the angle between the easy axis of the single-crystal Ni–Mn–Ga alloy and the applied magnetic field, and *θ* = the angle between the easy axis of the single-crystal Ni–Mn–Ga alloy and the magnetization). *E*_Zeeman_ = Zeeman energy, while *E*_MEA_ = magnetocrystalline anisotropic energy as shown by dotted curves. (**b**) Calculated shear stress for the MVR of the single-crystal Ni–Mn–Ga cube embedded in different porous silicone rubber matrices as a function of elastic modulus. The dotted horizontal line indicates the necessary shear stress required for the MVR of the single-crystal Ni–Mn–Ga alloy. Error bars are within the symbols. Please note that the shear stress in (**b**) was calculated based on the equations mentioned above.

To quantitively analyze the necessary shear stress (*τ*) required for the MVR of the single-crystal Ni–Mn–Ga cube, some calculations were conducted, and their results are discussed in this section. It is known that for the shape deformation of the composite materials, the necessary shear stress (*τ*) could be expressed as Equation (2) [38,39,43]:

(2) $$\tau =\frac{\Delta E}{s}$$

where *s* indicates the shear strain [43], while Δ*E* shows the energy difference of different two specific variants (i.e., Variant 1 and Variant 2). In the case of the 5M-martensite phase, the typical *c*/*a* is around 0.94 [44,45] and the shear strain (*s*) is reported to be approximately 0.12 [46]. The overall energy of each variant (*E_total_*) could be expressed using Equation (3) [38,39]:

(3) $$E_{total}=-\mu_{0}MHcos\;\left(\gamma -\theta \right)+K_{u}{sin}^{2}\theta$$

where *μ*_0_ is permeability, *M* is magnetization, *H* is magnetic field, *γ* is the angle between the easy axis of the single-crystal Ni–Mn–Ga alloy and the applied magnetic field, *θ* is the angle between the easy axis of the single-crystal Ni–Mn–Ga alloy and the magnetization, and *K*_u_ is the magnetic anisotropy constant. An illustration is shown in Figure 9a to reveal the relationships among the *M*, *H*, and easy axis (i.e., *c*-axis) of the Ni–Mn–Ga alloy. In this calculation, *K*_u_ was determined to be 165 kJ m^−3^ [47]. Here, the magnitude of the *H*-field was determined to commence the magnetic field-induced MVR of the single-crystal Ni–Mn–Ga alloy. The minimum energy could be obtained using Equation (4) [38,39]:

(4) $$\frac{\partial E}{\partial \theta }=-\mu_{0}MH\left(-cos\;\gamma sin\;\theta +sin\;\gamma cos\;\theta \right)+{2K}_{u}sin\;\theta cos\;\theta =0$$

A stable state in the variant is thus obtainable. Here, a magnetic field was applied to the <100>_p_ of the single-crystal Ni–Mn–Ga cube; hence, among the three variants, the *γ* of one variant is 0, while the *γ* of the two remaining variants is 90^o^. By using the aforementioned equations along with the parameters obtained from the literature [43,46,47], the shear stress could be estimated using Equation (1).

The estimated shear stress (*τ*) is shown in Figure 9b as a function of the elastic modulus of the porous silicone rubber. It was found that the shear stress required for the MVR of the single-crystal Ni–Mn–Ga cube embedded in the porous silicone rubber is in the range of around 1.0 to 1.3 MPa. It was found that while the elastic modulus of the silicone rubber is at approximately 1.15 MPa, the shear stress required for the MVR of the single-crystal Ni–Mn–Ga cube is around 1.0 MPa. This required shear stress is close to that required for the shear stress of the MVR of the single-crystal Ni–Mn–Ga cube, which is calculated to be about 0.9 MPa. Therefore, it is considered that by introducing a pore structure into the silicone rubber in order to lower its elastic modulus (i.e., in this case, around 1.15 MPa) and free the surface constraint, the necessary shear stress could be approached to the single-crystal specimen; hence, shape deformation is expected. Furthermore, meanwhile, the embrittlement of the single-crystal Ni–Mn–Ga alloy could also be solved using the porous silicone rubber. Additionally, it is also considered that besides the porous structure of the silicone rubber, by utilizing silicone rubber possessing an elastic modulus of around 1.15 MPa, a similar result could be achieved.

### 3.6. Comparison between the Solid and the Porous Structures of Silicone Matrix

According to the aforementioned results, it could be concluded that for attaining the magnetic field-induced MVR of the single-crystal Ni–Mn–Ga cube, two effective strategies could be carried out. The first strategy is to simply reduce the elastic modulus of the solid silicone rubber; while the second strategy is to introduce a certain porous structure to the silicone rubber matrix. By using these two methods, the MVR of the single-crystal Ni–Mn–Ga cube could be achieved relatively easily.

In the case of the utilization of various silicone rubbers with different elastic moduli, the magnetic field, which is necessary for the MVR of the single-crystal Ni–Mn–Ga cube, did not change much (Figure 6a); even the variation of the silicone rubber was in the window of about 4–5 MPa (see the large dashed rectangle in Figure 6a). On the other hand, by introducing a porous structure to the silicone rubber, a small difference in the elastic modulus of about 0.06–0.07 MPa (Figure 7) could have a great influence on the MVR. In the case of the porous structured silicone rubber, not only could the commencement of the MVR of the single-crystal Ni–Mn–Ga cube be reduced, but the accumulated magnetization change could also be enhanced.

Based on the above-mentioned things, it is considered that the porous structure of the silicone rubber could have a great influence on the MVR of the single-crystal Ni–Mn–Ga cube. It is believed that the small pores, which were generated by introducing the PFP to the silicone rubber, could reduce the constraint of the silicone rubber on the single-crystal Ni–Mn–Ga cube; that is, the MVR around the surface of the cube could be freed by introducing pore structures. A reduced effective elastic modulus could be achieved around the pores. Hence, the MVR of the single-crystal Ni–Mn–Ga cube could take place easily when there are some pores in the matrix. Thus, the MVR of the single-crystal Ni–Mn–Ga cube could be induced easily in the case of the porous structure with the elastic modulus of the matrix at around the same level.

### 3.7. Factors That Affect the MVR of the Single-Crystal Ni–Mn–Ga Alloy

#### 3.7.1. Back Stress from the Silicone Rubber Matrix

**Figure 10:**
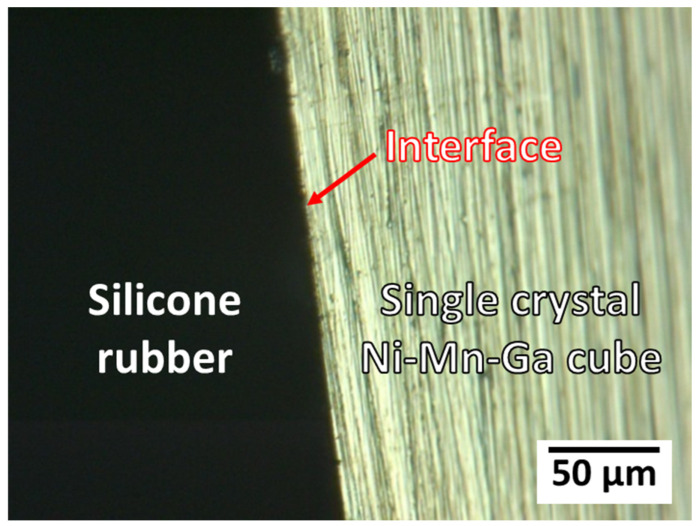
An optical image of the composite material of the single-crystal Ni–Mn–Ga cube/solid silicone rubber. The red arrow points to the interface of the single-crystal Ni–Mn–Ga cube and the solid silicone rubber.

Prior to the discussion of the factors that affect the MVR of the single-crystal Ni–Mn–Ga alloy, an optical image of the composite material is shown in Figure 10. It is confirmed that these two materials are well adhered to each other, without any gap between them. Therefore, with the introduction of a magnetic field, they undergo some interactions with each other. It is also necessary to mention that after the twinning motion, these two materials were still adhered to each other [37].

As an external magnetic field is applied to the single-crystal Ni–Mn–Ga alloy, magnetic field-induced shape deformation takes place and the single-crystal Ni–Mn–Ga alloy works on the silicone matrix. Therefore, the overall shape deformation of the composite material was achieved. Meanwhile, since the silicone rubber resists the shape deformation brought about by the single-crystal Ni–Mn–Ga alloy, back stress is generated from the silicone rubber and works on the single-crystal Ni–Mn–Ga alloy [35]. This back stress originating from the silicone rubber could be a factor restricting the MVR of the single-crystal Ni–Mn–Ga alloy. In addition, the greater the elastic modulus of the silicone rubber, the higher the back stress from the silicone rubber if the shape deformation amount of the single-crystal Ni–Mn–Ga alloy is the same.

#### 3.7.2. Volume Fraction of the Composite Materials

**Figure 11:**
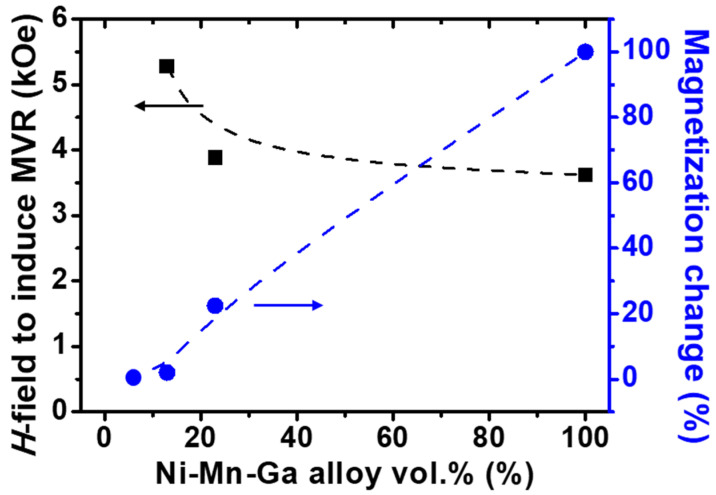
The necessary *H*-field required to induce MVR in the single-crystal Ni–Mn–Ga cube (left-hand-side *y*-axis with black square symbols) and the accumulated magnetization change brought about by the MVR of the single-crystal Ni–Mn–Ga cube (right-hand-side *y*-axis with blue circle symbols). Please note that the data were read and calculated from [36,37] and are plotted in this figure (error bars are within the symbols).

In the literature, the effect of the volume fraction of the composite materials has been reported [36,37]. In these reports, the volume fractions (vol.) of the single-crystal Ni–Mn–Ga cubes were about 7%, 13%, 23%, and 100%, respectively. Please note that 100 vol.% refers to a single-crystal Ni–Mn–Ga cube without any integration of the polymer matrix. In the observations, it was found that obvious MVR could be found in the *M*-*H* curves, as the vol.% of the single-crystal Ni–Mn–Ga cube was larger than 13%. That is to say, with 13 vol.% to 100 vol.% of the single-crystal Ni–Mn–Ga cube, MVR could be induced by an externally applied magnetic field. On the other hand, no apparent MVR could be found when the vol.% of the single-crystal Ni–Mn–Ga cube was less than 13 vol.% (i.e., the 7 vol.% specimens in references of [36,37]). Therefore, the critical vol.% could be determined to be at around 13 vol.% of the single-crystal Ni–Mn–Ga cube. In addition, the MVR of the single-crystal Ni–Mn–Ga cube could be induced when the external magnetic field was imposed at 5.28, 3.88, and 3.62 kOe for the 13 vol.%, 23 vol.%, and 100 vol.% composite materials, respectively (Figure 11).

Similar to the trend shown in the commencement of the MVR of the single-crystal Ni–Mn–Ga cube, the accumulated magnetization changes increased while the vol.% of the single-crystal Ni–Mn–Ga cube increased. Please note that the accumulated magnetization changes of the single crystal (100 vol.% single-crystal Ni–Mn–Ga cube) have been used as the reference of 100%, and the relative percentages of the composite materials are compared. It was found that in the 7 vol.% specimen, the accumulated magnetization change was, of course, 0%, while those of the 13% and 23% specimens were at about 2.0% and 22.4%, respectively (Figure 11).

According to the aforementioned results and discussions, firstly, the necessary magnetic field required for the commencement of the MVR and the accumulated magnetization change both depend on the volume fractions of the single-crystal Ni–Mn–Ga cube in the composite materials. Secondly, it was found that, in this work, when the silicone rubber has a non-porous structure (i.e., solid silicone rubber matrix), the elastic modulus hardly affects the commencement of the magnetic field-induced MVR (Figure 6a); however, the elastic modulus did affect the accumulated magnetization change (Figure 6b). Lastly, it was also observed in this study that the necessary magnetic field required for the commencement of the MVR and the accumulated magnetization change both depend on the elastic modulus of the porous silicone rubber (i.e., with the introduction of PFP). A brief summary of the aforementioned findings is shown in Table 2.

### 3.8. Deformation of the Composite Material

In estimating the deformations of the composites, the experiments described in Section 2.4.6 were conducted. The results regarding the deformations of the composite materials are shown in Table 3. According to the observations, it was found that the deformations of the composite materials showed a quite large displacement, originating from the MVR of a single-crystal Ni–Mn–Ga cube of about 22 to 25 μm, while the percentages of the deformations were about 0.60% to 0.75%, respectively. To compare the composites in this study and the Ni–Mn–Ga-based composites used in the literature, a table is shown as Table S1 in the Supplementary Materials [30,31,32,35,36,37,38,39].

## 4. Conclusions

In this study, various composite materials composed of single-crystal Ni–Mn–Ga cube/silicone rubber with or without the introduction of a porous structure using the PFP were fabricated and examined. Examinations of the mechanical properties of the pure solid/porous silicone rubber matrix materials were conducted and the analysis of the magnetic properties of the composite materials was also carried out. The important findings are summarized as follows:

1. According to the phase identification and the thermal analysis, the Ni–Mn–Ga cube was confirmed to be in the near-{100}_p_ single-crystal 5M-martensite phase with a ferromagnetism.
2. The higher the Hardness Shore A of the silicone rubber (i.e., from 23 to 60 of the Hardness Shore A), the higher the elastic modulus that was found, as expected.
3. With the introduction of pores to the silicone rubber by utilizing the PFP, the elastic modulus of the porous silicone rubber was successfully reduced in a good trend.
4. The obvious magnetic field-induced MVR of the single-crystal Ni–Mn–Ga cube was found in its *M*-*H* curve, and the observed necessary *H*-field required for the commencement of the MVR of the 5M-martensite Ni–Mn–Ga alloy at around 2 kOe corresponds well with the results in the literature.
5. The elastic modulus of the solid silicone rubber matrix hardly affected the necessary *H*-field required for the commencement of the MVR of the single-crystal Ni–Mn–Ga cube. However, the accumulated magnetization change depended on the elastic modulus of the solid silicone rubber matrix.
6. Both the necessary *H*-field required for the commencement of the MVR of the single-crystal Ni–Mn–Ga cube and the accumulated magnetization change are dependent on the elastic modulus of the porous silicone rubber matrix.
7. The configurations (vertical or parallel to the external *H*-field applied) of the pores in the composites used had a limited effect on the magnetic properties of the composite materials.
8. The displacement of around 22 to 25 μm of the composite materials could be obtained by taking advantage of the composite materials.

## Figures and Tables

**Table 1:** (a) Specification of all kinds of silicone rubbers and their corresponding curing agent catalysts used in this study. (b) The volume fraction (vol.%) of the silicone rubber (M8017 with Hardness Shore A of 50) and the PFP.

**(a) Silicone rubbers and the corresponding curing agent catalyst used in this study**						
Serial umber	M4400	M8520	SLJ3220	M8012	M8017	M4470
Hardness Shore A	23	27	35	45	50	60
Curing agent catalyst	T40	T40	T47	T40	T40	T40
Silicone:Catalyst (wt.%)	100:3	100:4	100:4	100:4	100:4	100:3
(**b) Volume percentage of the M8017 silicone rubber (Hardness Shore A = 50) and the PFP**						
M8017 silicone rubber (vol.%)	100	90	80	70	60	50
PFP (vol.%)	0	10	20	30	40	50

**Table 2:** The dependences of the necessary *H*-field required for the commencement of MVR and the accumulated magnetization change of the MVR of the single-crystal Ni–Mn–Ga cube on the (a) elastic modulus of solid silicone rubber matrix, (b) elastic modulus of porous silicone rubber, and (c) volume fraction of the single-crystal Ni–Mn–Ga alloy in the silicone rubber matrix [36,37].

Factor	Commencement of Magnetic Field-Induced MVR	Accumulated Magnetization Change	Reference
(a) Elastic modulus (solid)	X	O	This study
(b) Elastic modulus (porous)	O	O	This study
(c) Volume fraction of Ni–Mn–Ga alloy	O	O	[36,37]

**Table 3:** Measurements of the displacement of different composite materials with the silicone rubber possessing the elastic modulus of (a) 1.56 and (b) 1.95 MPa (i.e., silicone rubber with the Hardness Shore A values of 27 and 45), respectively.

Silicone Rubber Type	Elastic Modulus (MPa)	Displacement (μm)	Deformation (%)
(a) M8520 (Hardness Shore A = 27)	1.56	22	0.60
(b) M8012 (Hardness Shore A = 45)	1.95	25	0.75

## Data Availability

The data used are confidential.

## Supplementary Materials

The following supporting information can be downloaded at: https://www.mdpi.com/article/10.3390/mi14081604/s1, Figure S1. (a) Photo of the settings of the laser sensor measurement used for analyzing the deformation. (b) A zoomed-in figure of the stage is shown in the dashed line marked in (a). (c) An illustration of the laser sensor measurement setting used for analyzing the deformation. Figure S2. The M-H curves of the single crystal Ni-Mn-Ga cube in the composite materials triggered by an externally applied magnetic field as a function of elastic modulus of the various solid silicone rubber matrix. The examinations were conducted at RT under ambient with a magnetic field scan range from 0 to 10 kOe. (The numbers adjacent to the dots suggest the Hardness Shore A of the various silicone rubbers). Table S1. Comparison of the Ni-Mn-Ga alloys composed composites.
