# Supplementary material for: Investigations of Shape Deformation Behaviors of the Ferromagnetic Ni–Mn–Ga Alloy/Porous Silicone Rubber Composite towards Actuator Applications
Source: Micromachines (Basel). 2023 Aug 14;14(8):1604. doi: 10.3390/mi14081604 (PMC10456531; doi:10.3390/mi14081604)
Supplement: Supplementary file 1 [file micromachines-14-01604-s001.zip › micromachines-2526634-supplementary.docx]

**Supplemental Materials**

**Investigations of shape deformation behaviors of the ferromagnetic Ni-Mn-Ga alloy/porous silicone rubber composite towards actuator applications**

Wan-Ting Chiu*, Yui Watanabe, Masaki Tahara, Tomonari Inamura, and Hideki Hosoda

Institute of Innovative Research (IIR), Tokyo Institute of Technology,

4259 Nagatsuta–cho, Midori–ku, Yokohama 226–8503, Japan

*E-mail: chiu.w.aa@m.titech.ac.jp (for Wan-Ting Chiu)


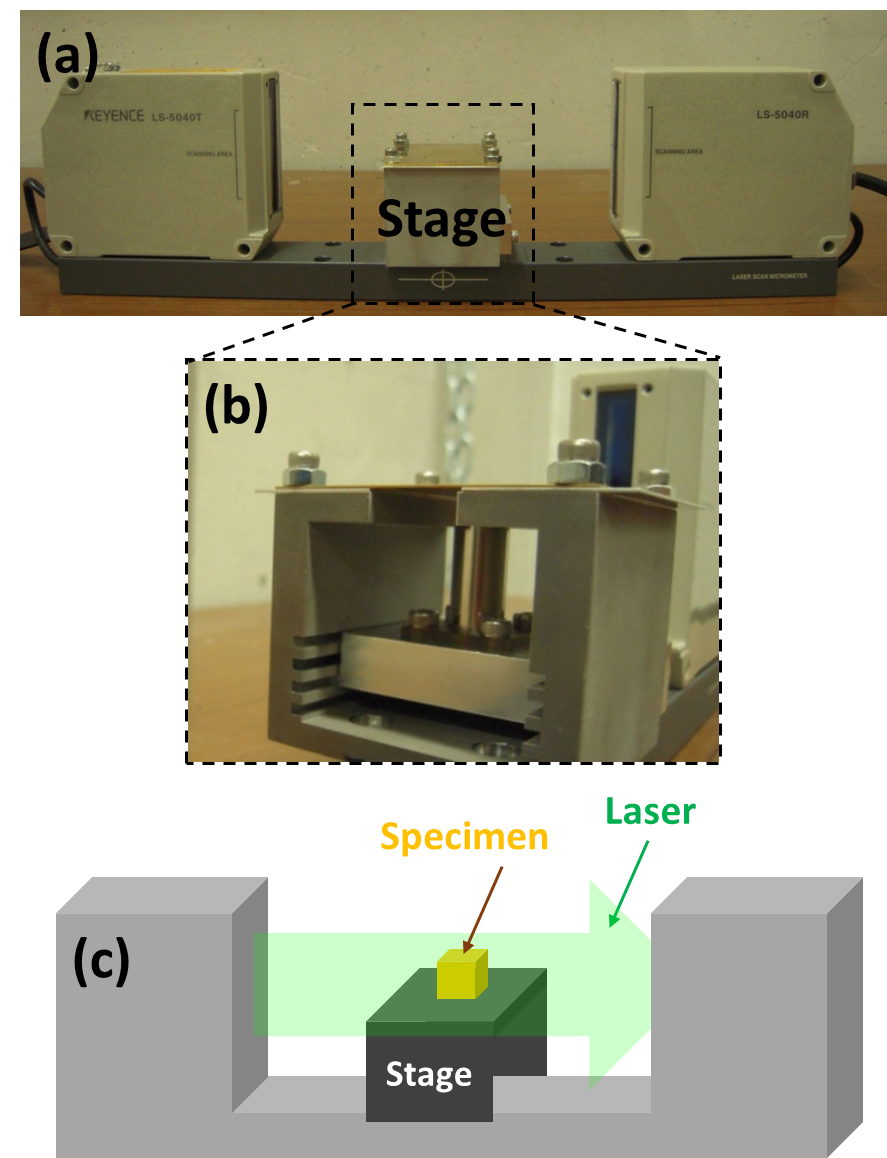


**Figure S1.** Figure S1. (a) Photo of the settings of the laser sensor measurement used for analyzing the de-formation. (b) A zoomed-in figure of the stage is shown in the dashed line marked in (a). (c) An illustration of the laser sensor measurement setting used for analyzing the deformation.


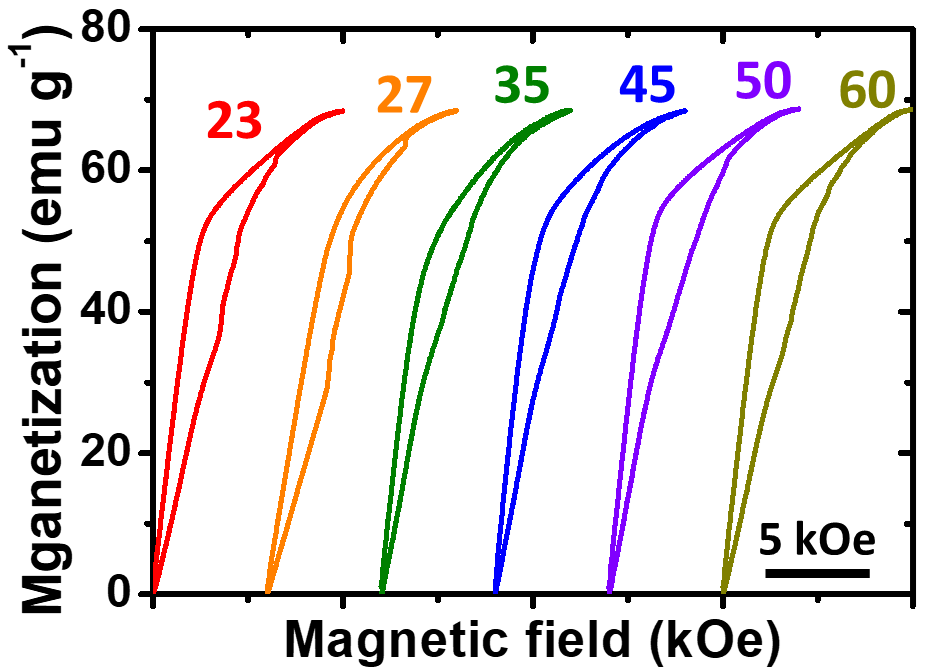


**Figure S2.** The *M*-*H* curves of the single crystal Ni-Mn-Ga cube in the composite materials triggered by an externally applied magnetic field as a function of elastic modulus of the various solid silicone rubber matrix. The examinations were conducted at RT under ambient with a magnetic field scan range from 0 to 10 kOe. (The numbers adjacent to the dots suggest the Hardness Shore A of the various silicone rubbers.)

**Table S1.** Comparison of the Ni-Mn-Ga alloys composed composites.

| **Specimen** | **Apparent phase** | **Applied field** | **Deformation strain** | **Others** | **Ref.** |
| --- | --- | --- | --- | --- | --- |
| SC Ni-Mn-Ga cube/silicone | 5M martensite | Magnetic field | ~4% (macro) | •30 vol.% particles | [35] |
| SC Ni-Mn-Ga cube/silicone | 5M martensite (major) +  7M martensite (minor) | Compression | ~3.5% (5M martensite)  ~5.5% (7M martensite)  (micro strains) | •20 vol.% particles | [30] |
| SC Ni-Mn-Ga cube/silicone | 5M martensite | Magnetic field | -- | •7 vol.% cube  •13 vol.% cube  •23 vol.% cube | [37] |
| SC Ni-Mn-Ga cube/silicone | 5M martensite (major) +  7M martensite (minor) | Magnetic field | ~4% (macro) | •20 vol.% particles | [31] |
| SC Ni-Mn-Ga cube/silicone | 7M martensite | Magnetic field | -- | •Opened surface one-cube (10 vol.% Ni-Mn-Ga alloy) | [36] |
| SC Ni-Mn-Ga cube(s)/epoxy | 5M martensite | Compression/ Magnetic field | ~3.6% (by *σ*-field)  ~0.26% (by *H*-field) | •One-cube (30 vol.% Ni-Mn-Ga alloy) | [39] |
| SC Ni-Mn-Ga cube(s)/silicone | 5M martensite | Compression/ Magnetic field | 2%~3% (by *σ*-field) | •One-cube (30 vol.% Ni-Mn-Ga alloy)  •Two-cube (20 vol.% Ni-Mn-Ga alloy)  •Two-cube (25 vol.% Ni-Mn-Ga alloy)  •Two-plate (50 vol.% Ni-Mn-Ga alloy) | [39] |
| SC Ni-Mn-Ga particles/Cu foil | 5M martensite | Magnetic field | 2.7% (micro)  ~3% (macro) | Sandwich structure  (layered composite) | [32] |
| SC Ni-Mn-Ga particles/epoxy | 5M martensite | Compression/ Magnetic field | 2% | •One-particle  •10 vol.% particles  •40 vol.% particles | [38] |
| SC Ni-Mn-Ga alloys/silicone | 5M martensite | Compression/ Magnetic field | -- | •10 vol.% particles  •20 vol.% particles  •40 vol.% particles | [38] |
| SC Ni-Mn-Ga alloys/porous silicone | 5M martensite | Magnetic field | ~0.60 (macro)  ~0.75 (macro) | •6.5 vol.% cube (Hardness Shore A silicone rubber = 27)  •6.5 vol.% cube (Hardness Shore A silicone rubber = 45) | This study |
